# Single-nuclei transcriptomics reveals TBX5-dependent targets in a patient with Holt-Oram syndrome

**DOI:** 10.1172/JCI180670

**Published:** 2024-11-14

**Authors:** Jeffrey D. Steimle, Yi Zhao, Fansen Meng, Mikaela E. Taylor, Diwakar Turaga, Iki Adachi, Xiao Li, James F. Martin

**Affiliations:** 1 Department of Integrative Physiology, Baylor College of Medicine, Houston, Texas, USA.; 2 McGill Gene Editing Laboratory, Texas Heart Institute, Houston, Texas, USA.; 3 Department of Pediatrics, Baylor College of Medicine, Houston, Texas, USA.; 4 Division of Critical Care Medicine, Texas Children’s Hospital, Houston, Texas, USA.; 5 Department of Surgery, Baylor College of Medicine, Houston, Texas, USA.; 6 Division of Congenital Heart Surgery, Texas Children’s Hospital, Houston, Texas, USA.; 7 Cardiomyocyte Renewal Laboratory, Texas Heart Institute, Houston, Texas, USA.; 8 Center for Organ Repair and Renewal, Baylor College of Medicine, Houston, Texas, USA.

**Keywords:** Cardiology, Genetics, Cardiovascular disease, Genetic diseases, Molecular genetics

## To the Editor:

Holt-Oram syndrome (HOS), characterized by heart and forelimb defects, is caused by mutations in the T-box transcription factor TBX5 (1). While much has been done to elucidate the transcriptional mechanisms of TBX5 in model systems (2–5), transcriptomics of tissue from patients with HOS is lacking. Here, we report a rare opportunity to interrogate the cardiac transcriptome of HOS using high-resolution, single-nucleus transcriptomics (snRNA-Seq) of left ventricular (LV) tissue from a 10-year-old female patient with HOS undergoing transplant surgery.

**Figure 1:**
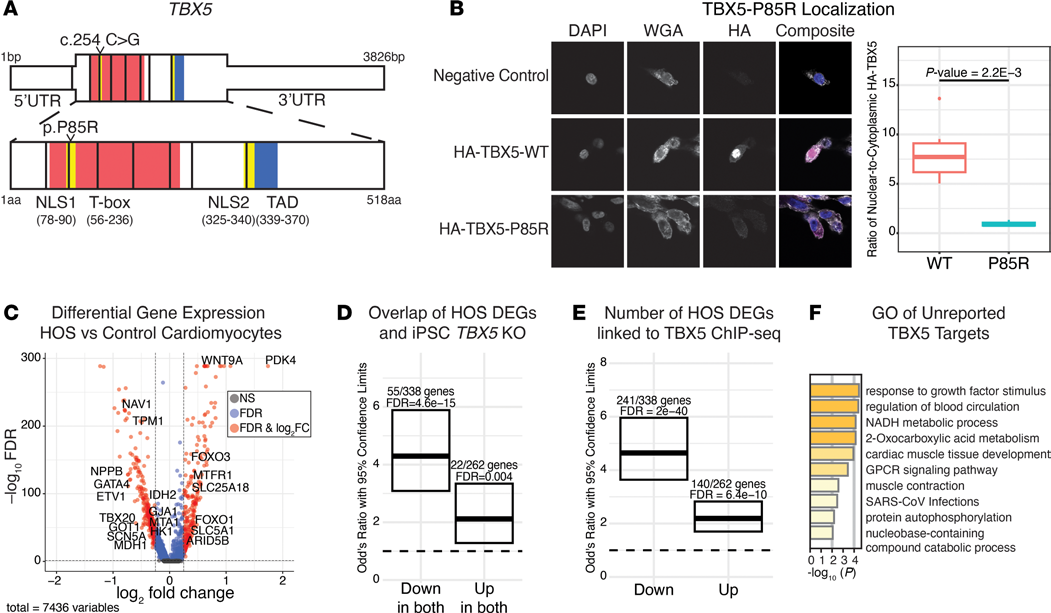
Identification of TBX5-dependent targets at single-cell resolution. (**A**) *TBX5* gene (top) and protein (bottom) with domains labeled. Patient mutation c.254C>G (p.P85R) is indicated by an arrowhead. (**B**) Anti-HA and wheatgerm agglutinin (WGA) immunofluorescence staining of FaDu cells transfected with HA-TBX5-WT or HA-TBX5-P85R (original magnification, ×40). Ratiometric quantification of nucleus-to-cytoplasm HA signal by box plot (*n =* 6). *P* value was determined by Welch’s 2-sample *t* test. (**C**) Volcano plot showing the distribution of differentially expressed genes (FDR <0.05 and |log_2_fold change| >0.25) comparing HOS and control cardiomyocytes. (**D**) OR by Fisher’s exact test comparing the overlap of down- and upregulated genes identified in **C** and in published TBX5-KO iPSC-derived cardiomyocytes (2). (**E**) OR by Fisher’s exact test comparing the overlap of down- and upregulated genes identified in **C** and published TBX5 ChIP-Seq from iPSC-derived cardiomyocytes (Supplemental Ref 14). (**F**) Gene ontology (GO) term analysis of *TBX5*-dependent genes associated with TBX5 ChIP-Seq not previously reported in cardiomyocyte-derived iPSCs or mouse tissue.

The patient presented with atrial and ventricular septal defects, right thenar hypoplasia, and sick sinus syndrome (Supplemental Figure 1A; supplemental material available online with this article; https://doi.org/10.1172/JCI180670DS1). The patient underwent genetic testing, revealing coding mutations in TBX5 (p.P85R), DNHD1, and ZNF469 (Supplemental Table 1). While phenotypically characteristic of HOS, DNHD1 variants coincide with laterality defects and may contribute to the pathology (6). The TBX5 mutation affects a conserved proline residue in a nuclear localization signal (Figure 1A and Supplemental Figure 1, B and C). Structural predictions suggest that, while nonpolar, cyclic proline-85 is interposed between nonpolar ring structures in adjacent helices, charged arginine-85 presents on the surface where it may disrupt surface interactions (Supplemental Figure 1D). In cell culture, TBX5-P85R was predominantly cytoplasmic compared with the reference allele (Figure 1B) and showed weaker expression (Supplemental Figure 1E), suggesting a mechanism for this loss-of-function allele (1).

The patient with HOS had a left coronary artery obstruction in the setting of pulmonary artery band removal and ventricular septal defect closure requiring revascularization, leading to ventricular insufficiency, heart failure, and indication for transplantation. Explanted LV tissue was collected, and snRNA-Seq was performed. As TBX5 is predominantly expressed in cardiomyocytes (Supplemental Figure 1F), we performed differential expression testing comparing HOS and matched nonfailing donor cardiomyocytes, identifying 338 downregulated and 262 upregulated genes (Figure 1C and Supplemental Table 2). These genes are associated with contraction and conduction (3), known functions of TBX5 (Supplemental Figure 1G). We compared these genes with reported TBX5 targets from induced pluripotent stem cell–derived (iPSC-derived) cardiomyocytes and mouse models (2, 3). While there was an appreciable overlap among the datasets (122 of 600 with iPSC-derived cardiomyocytes and 229 of 600 with mouse atrial knockout), 143 of 338 downregulated genes were newly identified (Figure 1D and Supplemental Figure 1H).

We used TBX5 ChIP-Seq of iPSC-derived cardiomyocytes to identify direct targets. Two-thirds of the downregulated and half of the upregulated genes were associated with TBX5 binding (Figure 1E). Of the downregulated genes, 80 were not in previous datasets, including MTA1, a gene found to genetically and physically interact with TBX5 in ventricular development. One-fifth of these direct TBX5 targets are associated with metabolism (Figure 1F), including glucose and glycogen metabolism (HK1 and GYS1), TCA cycle (IDH2 and MDH1), and amino acid synthesis (GOT1 and BCKDHB). TBX5 regulation of metabolism is underexplored and warrants further investigation.

To distinguish between the effects of TBX5-P85R and heart failure, we compared our gene lists with those of published pediatric and adult cardiomyopathies. We found that 28% of the downregulated genes were not changed or upregulated in cardiomyopathy, with 78% being predicted direct targets, including several of the metabolic pathway genes (Supplemental Figure 1I). Conversely, 90% of the upregulated genes were also upregulated in cardiomyopathy.

Altogether, the HOS patient transcriptome reveals undescribed TBX5 transcriptional targets, while sharing many features with HOS models and furthering our understanding of HOS pathophysiology. This exciting addition to the growing body of pediatric cardiovascular datasets benefits the wider community by offering a rare opportunity to study the HOS transcriptome firsthand.

## Supplementary Material

Supplemental data

Unedited blot and gel images

Supplemental table 1

Supplemental table 2

Supporting data values

## Version 1 11/14/2024

In-Press Preview

## Version 2 01/16/2025

Electronic publication
