# Supplementary material for: Single-nuclei transcriptomics reveals TBX5-dependent targets in a patient with Holt-Oram syndrome
Source: J Clin Invest. 2024 Nov 14;135(2):e180670. doi: 10.1172/JCI180670 (PMC11735084; doi:10.1172/JCI180670)
Supplement: Supplemental data [file jci-135-180670-s122.pdf]

SUPPLEMENTAL MATERIALS: Holt-Oram syndrome at single cell resolution

Authors and Affiliations

Jeffrey D. Steimle[1], Yi Zhao[2], Fansen Meng[2], Mikaela E. Taylor[1], Diwakar Turaga[3,4], Iki Adachi[5,6], Xiao Li[2], and James F. Martin[1,2,7,8]

1. Department of Integrative Physiology, Baylor College of Medicine, Houston, TX, USA
2. McGill Gene Editing Laboratory, Texas Heart Institute, Houston, TX, USA
3. Department of Pediatrics, Baylor College of Medicine, Houston, TX, USA
4. Division of Critical Care Medicine, Texas Children's Hospital, Houston, TX, USA
5. Department of Surgery, Baylor College of Medicine, Houston, TX, USA
6. Division of Congenital Heart Surgery, Texas Children's Hospital, Houston, TX, USA
7. Cardiomyocyte Renewal Laboratory, Texas Heart Institute, Houston, TX, USA
8. Center for Organ Repair and Renewal, Baylor College of Medicine, Houston, TX, USA

Address correspondence to: James Martin, Baylor College of Medicine, One Baylor Plaza, Houston, Texas 77030, USA. Phone: +1-713-798-5931; Email: jfmartin@bcm.edu.

ADDITIONAL MAIN TEXT REFERENCES

7. Collavoli A, et al. TBX5 nuclear localization is mediated by dual cooperative intramolecular signals. *J Mol Cell Cardiol*. 2003;35(10):1191–1195.

8. Sim CB, et al. Sex-Specific Control of Human Heart Maturation by the Progesterone Receptor. *Circulation*. 2021;143(16):1614–1628.

9. Koenig AL, et al. Single-cell transcriptomics reveals cell-type-specific diversification in human heart failure. *Nat Cardiovasc Res*. 2022;1(3):263–280.

10. Hill MC, et al. Integrated multi-omic characterization of congenital heart disease. *Nature*. 2022;608(7921):181–191.

11. Bruneau BG, et al. A murine model of Holt-Oram syndrome defines roles of the T-box transcription factor Tbx5 in cardiogenesis and disease. *Cell*. 2001;106(6):709–721.

12. Nadadur RD, et al. Pitx2 modulates a Tbx5-dependent gene regulatory network to maintain atrial rhythm. *Sci Transl Med*. 2016;8(354):354ra115.

13. van Ouwerkerk AF, et al. Patient-specific TBX5-G125R variant induces profound transcriptional deregulation and atrial dysfunction. *Circulation*. 2022;145(8):606–619.

14. Ang Y-S, et al. Disease Model of GATA4 Mutation Reveals Transcription Factor Cooperativity in Human Cardiogenesis. *Cell*. 2016;167(7):1734–1749.e22.

15. Waldron L, et al. The Cardiac TBX5 Interactome Reveals a Chromatin Remodeling Network Essential for Cardiac Septation. *Dev Cell*. 2016;36(3):262–275.

METHODS AND SUPPLEMENTAL INFORMATION

Research Ethics for Donated Tissues

Cardiac tissues samples used in this study were collected during cardiothoracic surgeries performed at Texas Children's Hospital (Houston, Texas). The protocols for the procurement and use of these patient samples were approved by the Institutional Review Board for Baylor College of Medicine and Affiliated Hospitals (Protocol Number H-26502). With the help of the Heart Center Biorepository at Texas Children's Hospital, consent was obtained from patients undergoing repeat heart transplantation. The anatomic location of tissue collected is the left ventricle apex.

Patient Clinical History

At 7 months of age, the patient was diagnosed with a high secundum atrial septal defect, multiple apical-to-midventricular muscular ventricular septal defects, and right thenar hypoplasia (**Supplemental Figure 1A**). A pulmonary artery band was placed at 8 months of age. Post-surgical course was complicated by sick sinus syndrome requiring pacemaker placement. At 3 years of age, the patient underwent catheter ASD and VSD closure. Pulmonary artery band removal led to aortic bleeding and a brief ECMO course. On evaluation, the left coronary artery was occluded requiring surgical revascularization. Over the next 7 years, the patient had ongoing depressed cardiac function and worsening heart failure symptoms. At 10 years of age, the patient underwent orthotopic heart transplantation at Texas Children's Hospital at which time, explanted tissue was collected.

Patient Genetics

Based on patient phenotype, targeted sequencing of *TBX5* was performed and identified the patient is heterozygous for c.254C>G (p.P85R, **Supplemental Figure 1B**). The patient and parents also underwent whole exome trio sequencing, confirming the de novo mutation in *TBX5*. Whole exome sequencing also identified mutations in *DNHD1*, c.4732_4733delinsCG (p.A1578R) and c.6739T>C (p.Y2247H), both of which are predicted to be tolerated (**Supplemental Table 1**). The patient inherited the first allele from the father and the second allele from the mother. *DNHD1* is a cilia-related protein primarily described in the context of male infertility and sperm motility defects, including in homozygous mutations in mouse models (1). However, there is a recent report of laterality defects attributed to nonsense and frameshift mutations of *DNHD1* that may contribute to structural heart defects (2). Also identified were mutations in *ZNF469*, a transcriptional regulator of extracellular matrix proteins associated with Brittle cornea syndrome (3, 4), specifically c.745G>A (p.G249R), c1742C>T (p.P581L), and c.10241G>C (p.R3414T), with

the first mutation in the maternal allele and the second and third mutations in the paternal allele. The mother and father were heterozygous for the respective alleles. Based on our review of the literature, there have been no reported links of *ZNF469* to heart development. Additionally, the International Mouse Phenotyping Consortium (IMPC) reports no cardiac phenotypes for homozygous mutants of *Znp469*, the mouse *ZNF469* homologue, despite other organ problems including ocular defects (https://www.mousephenotype.org/) (5, 6).

Sex as a biological variable

For direct comparisons with the female HOS patient, the non-failing donors were all female, aged 9.5 to 11, to minimize sex- and age-matched differences. The heart failure datasets include both male and female samples.

Protein Alignment

T-box protein alignment was performed using UniProt Align tool (**Supplemental Figure 1C**). T-box sequences used were RefSeq accession: NP_000183.2 (TBX5), NP_005985.3 (TBX2), NP_005987.3 (TBX3), NP_060958.2 (TBX4), NP_004599.2 (TBX6), NP_001157745.1 (MGA), NP_006584.1 (TBR1), NP_001265111.1 (EOMES), NP_037483.1 (TBX21), NP_005140.1 (TBX19), NP_003172.1 (TBXT), NP_542377.1 (TBX1), NP_005986.2 (TBX10), NP_001317606.1 (TBX15), NP_001073977.1 (TBX18), NP_001071121.1 (TBX20), and NP_058650.1 (TBX22). Dendrogram relationships were adopted from published work (7).

AlphaFold 3 Predictions and PyMOL Analysis

We performed protein folding predictions on the human reference TBX5 or TBX5-Pro85Arg along with double stranded DNA representing the DNA binding motif (dsDNA-TAAGGTGTGAG) using AlphaFold 3 through AlphaFold Server using default settings (8). Comparing the 5 outputs for each of the peptides, the predicted structures were all similar, especially the T-box domain, and matched the published crystal structures of TBX5 (9, 10). We focused our interest on the region of Pro85/Arg85. First, we find that in the wildtype peptides (with or without DNA), the ring structure of Pro85 is interposed between the ring structures of Phe84 and Trp64. Trp64, Phe84, and Pro85 are highly conserved residues present in all human T-box proteins. Given the charged nature of arginine, PyMOL was used to predict polar interactions of mutant Arg85. Arg85 is predicted to interact with Asn229 and Glu60. We next asked whether Asn229 or Glu60 are predicted to interact with any other residues in the TBX5-Pro85Arg or TBX5-WT structures and found that an interaction between Glu60 and Lys88 present in the WT but absent in the TBX5 mutant. Lastly,

as this region has been described as one of two nuclear localization signals present in TBX5, we examined the surface charges of the WT and Pro85Arg mutant. We found that in the WT, Glu60 and Lys88 are in close proximity, and a pocket is present over the site of the Pro85. However, in the mutant, Arg85 and Glu60 interact, filling the pocket and leaving negatively charged Lys88 unpartnered on the surface. Models were overlaid and images (**Supplemental Figure 1D**) were generated using PyMOL v2.5.5.

Cell culture, Antibody Staining, and Western Blots

N-terminally HA-tagged wildtype human TBX5 or TBX5-Pro85Arg were cloned into a pUBC backbone. The N-terminal HA tag is separated from TBX5 by a short poly-GS linker. Negative control plasmid is the backbone with no insert.

For immunofluorescence, each plasmid (20ng per well) was transfected into low confluency FaDu (ATCC: FaDu HTB-43), a human squamous cell carcinoma line, using Lipofectamine 3000 (Thermo Fisher Scientific) on 8-well chamber slides. After 48 hours, cells were fixed and incubated with anti-HA antibody (Cell Signaling Technology, C29F4, rabbit mAb). Cells were also treated with wheat germ agglutinin (WGA) and DAPI prior to imaging. Imaging was performed using a Zeiss LSM 780 NLO Confocal/2-Photon Microscope (Baylor College of Medicine, Optical Imaging & Vital Microscopy Core). Nuclear-to-cytoplasmic HA signal was quantified in imageJ using the signal found in the nucleus (stained by DAPI) divided by the signal found in the cytoplasm (WGA bounded). Statistics and graphing performed in R using Welch's two sample T-test.

For Western blotting, negative control plasmid and either the wildtype TBX5 or TBX5-Pro85Arg expression constructs were transfected using Lipofectamine 2000 into low confluency (~30%) HeLa cells (ATCC: HeLa CCL-2) in a 6-well plate. Plasmids were transfected in a gradient: 100ng, 200ng, or 400ng. After 36 hours, cells were lysed using RIPA lysis buffer and protein collected. Protein was divided for whole cell lysate (WCL) and immunoprecipitation (IP). For IP, magnetic anti-HA beads from Thermo Scientific (88837) were used to enrich the HA-tagged peptides. Western Blot was performed using anti-HA antibody (Cell Signaling Technology, C29F4, rabbit mAb).

Publicly Available Data

Previously published cardiac snRNA-seq data from pediatric donors were obtained from the NIH Gene Expression Omnibus (GEO) as non-failing controls. These datasets include three libraries from donor "UK1" reported by Hill et al. (GSE203275), one library from donor "Young2" by Sim et

al. (GSE156707), and one library from donor "13_235" by Koenig et al. (GSE183852) (11-13). All samples originated from the left ventricles of female donors, with ages ranging from 9.5 to 11 years old. The following publicly available data was also used this work: *TBX5*-dependent gene lists from iPSC single cell RNA-seq, Table S4, GEO GSE137876 (14); *Tbx5*-dependent gene lists from mouse ventricle, GEO GSE125823 (15) and mouse atria, GEO GSE129503 (16) and GEO GSE167082 (17); TBX5 ChIP-seq from iPSC, GEO GSM2280011/GSE85631 (18); pediatric hypertrophic cardiomyopathy (pHCM), pediatric dilated cardiomyopathy (pDCM), and adult dilated cardiomyopathy (aDCM), GEO GSE203275/ GSE183852 (11, 13); and promoter capture Hi-C, ArrayExpress accession no. E-MTAB-6014 (19).

Sample Collection and Nuclear Isolation

Cardiac tissue was collected in the operating room during heart transplantation. Cardiac tissue samples were kept in cold saline on ice during transfer to the laboratory for preservation. Cardiac tissue samples were carefully dissected into multiple aliquots, which were flash-frozen and stored at –80 °C Nuclear isolation was performed as described previously (11). Briefly, frozen cardiac tissue was dissociated by using a Dounce homogenizer. Single nuclei were isolated via fluorescence-activated cell sorting (FACS).

Single-nucleus RNA Sequencing

SnRNA-seq was performed by using the 10X Genomics platform. Isolated nuclei and cells were loaded into the 10X Genomics Chromium Controller to obtain the gel beads in emulsion. The sequencing libraries were then prepared according to the manufacturer's protocols for the Single-cell 3' Reagents Kits v3. Sequencing was performed by using the NovaSeq 6000 systems.

snRNA-seq Data Processing and Integration

All newly generated and published snRNA-seq datasets were processed using a uniformed pipeline described previously (20). Briefly, raw sequencing reads were aligned to the genome (build GRCh38) using the 10X Genomics toolkit CellRanger version 7.1.0 (cellranger count) with --include-introns set to true. All other parameters were left as defaults. Quality control metrics generated by CellRanger were inspected for each library. To remove background signals from ambient transcripts, the raw UMI count matrices were further processed by CellBender version 0.2.2 (cellbender remove-background) with --total-droplets-included = 25,000, --low-count-threshold = 15, and --epochs = 200. To minimize the loss of valid cell barcodes called by CellRanger, we also set --expected-cells at 1.5 times of CellRanger output nuclei number. The

output matrices from CellBender were filtered to only include valid cell barcodes that were also identified by CellRanger. Additional quality controls at single nucleus level were performed for each library. We first identified low-quality nuclei based on fixed cut-offs of UMI count per nucleus > 200, gene count per nucleus > 150 and mitochondria gene-derived UMI < 5%. Around 18.03% of total nuclei failed these hard cut-offs and were removed from the data sets. Then, a set of dynamic cut-offs based on per-library data distribution were calculated, which is essential to account for heterogeneity between samples. For each library, an upper and lower bound were set at the 75th percentile plus 1.5 times the interquartile range (IQR) and the 25th percentile minus 1.5 times IQR, respectively, for UMI count and gene count per nuclei. Around 7.6% of total nuclei were outside of the upper and lower bounds and were removed from the data sets. Next, the remaining 54,396 nuclei were evaluated by the Scrublet tool to identify potential doublets, with parameters expected_doublet_rate = 0.15 and call_doublets threshold = 0.25 (21). Around 12.1% nuclei were labeled as potential doublets but were kept in the data set. Finally, we integrated all samples and corrected batch-effect using a deep generative models scANVI (22). The scANVI model was trained with the Litviňuková, M. et al data set as an annotated reference (23). Fifty dimensions of the scANVI latent space was reduced to generate the final global UMAP for 2-D visualization.

Clustering and Annotation

We applied FindNeighbors function of the Seurat version 4.2.0 package to generate the shared nearest-neighbor graph (SNN) using the scANVI latent space (24). We defined clusters based on the SNN using Louvain algorithm with an optimized resolution of 1.0. As the scANVI model was trained with the Litviňuková, M. et al data set as a reference (23), we examined the predict cell type identities for each cell cluster. Based on both scANVI predicted labels and the expression of known cardiac cell type marker genes, we labeled all main clusters, except for one minor cluster enriched with mitochondria gene expression as an ambiguous cluster. Subsequent re-clustering of each major cell type was performed using an iterative approach. Within each major cell type, subclusters enriched with previously called doublets were examined for the expression of main cell type marker genes and were collectively labeled as ambiguous cells. Only the non-ambiguous nuclei were used for subsequent analyses. To visualize the subtle heterogeneity within the cardiomyocyte and cardiac fibroblast cell populations, a new neighborhood graph was constructed based on scANVI latent dimensions for each cell type. The optimal resolutions for subclustering were determined by clear separation in the UMAP dimension, robust identification of >30 significantly differentially expressed genes across subclusters.

Downstream Analysis of snRNA-seq

Differential gene expression of HOS and control cardiomyocytes was performed using the FindMarkers function from Seurat (24). A gene was determined as significant if it appeared in 10% of nuclei of either HOS or control samples, had a log2-fold change greater than 0.25, and an FDR < 0.05. For overlaps with the public ChIP-seq data, the ChIPpeakAnno package function annotatePeakInBatch was used to annotate each peak to the closest two promoters in both directions (25, 26) or using published associations of cis-regulatory elements to promoters from promoter capture Hi-C of iPSC-derived cardiomyocytes (19). For overlap with TBX5 knockout iPSC-derived cardiomyocytes (14), the gene list was used as published. For overlap with mouse atria and ventricles (15-17), an FDR less than 0.05 was used to determine significance. Significance of overlaps was calculated using a Fisher's Exact Test in R and multiple testing correction was performed using False Discovery Rate. Gene ontology (GO) analysis was performed using Metascape (27).

Expression of *TBX5* in Ventricular Cardiomyocytes

We detect *TBX5* mRNA in 45.0% of the left ventricular cardiomyocytes in our dataset. While not as abundant as cardiomyocyte transcription factors like *MEF2A* or *MEF2C* (92.8% and 77.7%, respectively), it is still more abundant than other cardiomyocyte transcription factors reportedly decreased after development such as *NKX2-5* (13.5%). Furthermore, in the cells in which *TBX5* is detected, the expression level is comparable to that of *MEF2C* (~0.02% of total reads). Additionally, the human protein atlas (https://www.proteinatlas.org/) (28, 29) reports strong nuclear expression and staining of TBX5 protein in adult ventricular cardiomyocytes (50–64-year-old hearts). Together, this indicates that TBX5 is still expressed in and likely plays an active transcriptional role in pediatric left ventricular cardiomyocytes.

Ability to Detect Changes Across Datasets for Novel HOS Genes

To address a possible cut-off issues between various datasets, we examined the published datasets with both significant and non-significant datapoints reported, i.e., *Tbx5* knockout atria (16), *Tbx5* knockout ventricle (15), and $Tbx5^{G125R}$ left and right atria (17). We correlated the gene expression changes for the genes not previously identified in a pairwise fashion. We found that there was no significant correlation between any of these datasets and the HOS dataset for the novel genes (*Tbx5* knockout atria: Spearman's ρ = 0.039, p-value = 0.71; *Tbx5* knockout ventricle: Spearman's ρ = -0.085, p-value = 0.42; $Tbx5^{G125R}$ left atria: Spearman's ρ = 0.019, p-value = 0.86;

and *Tbx5*^{G125R} right atria: Spearman's ρ = 0.076, p -value = 0.47). We took an alternative approach and asked whether a subset of the genes showed a similar trend across the published datasets. To do this, we binarized the fold-changes (greater than or less than 0). By this method, we found that 15/63 novel down-regulated and 7/28 novel up-regulated genes showed a similar trend across 3 or more datasets. From this, we do not believe statistical cut-offs was the primary reason for identification of new TBX5-dependent genes.

Given the confounding factor of heart failure, we next focused on the 75 down-regulated genes that are predicted to be direct targets of TBX5 and less likely to be due to heart failure (**Supplemental Table 2 and Supplemental Figure 1I**). We identified that 32/75 (43%) down-regulated genes have been identified as down-regulated in one or more of the published datasets. If we do a similar analysis with down-regulated direct targets of TBX5 irrespective of heart failure overlap, we find a similar proportion, 44% (106/241), were also previously identified. This suggests that the heart failure background had little effect for at least the down-regulated genes.

Statistics Summary

- Imaging quantification (Figure 1B): statistics and graphing performed using Welch's two sample T-test with two-tails.
- Differentially expressed genes (Figure 1C): statistics performed using the Wilcoxon Rank Sum test on all genes with a percent expression greater than 10% of cells.
- Enrichment analyses (Figures 1D, 1E, S1H, and 1SI): statistics performed using the Fisher's exact test.

Data Availability

The raw sequence files, raw expression matrices, and processed single-cell object files were deposited to GEO, GSE261014.

Acknowledgements

This project was supported by grants from the NIH through R01HL127717, R01HL118761, R01HL171574, and R01HL169511 to JFM; K99HL169742 and F32HL156465 to JDS. JFM was supported by the Vivian L. Smith Foundation. YZ and XL were supported by the Don McGill Gene Editing Laboratory of the Texas Heart Institute. MET was supported by the Baylor College of Medicine SMART Program. Special thanks to Paul Swinton at the Texas Heart Institute for assisting with experimentation.

Supplemental Figure 1

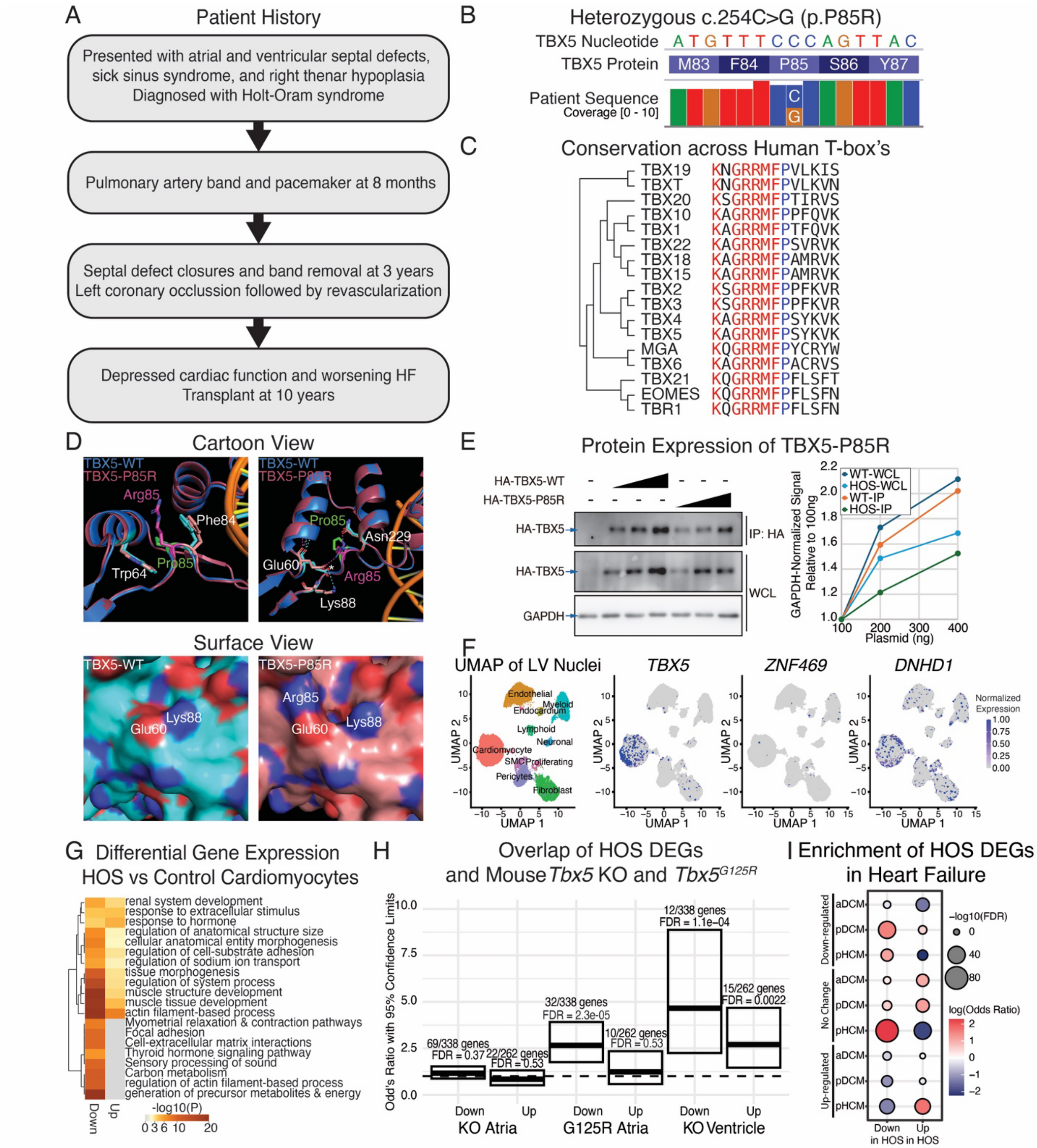

Supplemental Figure 1.

A. Chronological highlights of major medical events in the HOS patient's history.
B. Coverage tracks from the HOS patient snRNA-seq showing heterozygous *TBX5* c.254C>G.
C. Alignment of all human T-box transcription factors surrounding TBX5 proline 85 (blue). This proline is present in all described human T-box factors, and is present in a region of relatively high conservation. Amino acids labeled in red are likewise present in all human T-box factors. Dendrogram is based on published work (7).
D. Overlay of both AlphaFold3 prediction models as PyMOL Cartoon for wildtype TBX5 (blue) and TBX5-P85R (pink) on top. Proline 85 (green) and Arginine 85 (pink) are highlighted along with Tryptophan 64 (upper left), Phenylalanine 84 (upper left), Glutamic acid 60 (upper right), Lysine 88 (upper right), and Asparagine 229 (upper right) in white. The white asterisk highlights the predicted change in interaction between Glu60 and Lys88 (blue dashed line) to Glu60 and Arg85 (pink dashed line). This predicted change in interaction can also be seen in the surface view (bottom).
E. Western blots for the immunoprecipitation (IP) and whole cell lysate (WCL) of anti-HA of HeLa cells transfected with HA-TBX5-wildtype (WT) or HA-TBX5-P85R (left). Anti-GAPDH is used as loading control. Increasing concentrations of plasmid were provided to the cells with the doses representing 100ng, 200ng, and 400ng of total plasmid transfected. Ratiometric quantifications using the GAPDH loading control as a standard and normalized relative to 100ng of either wildtype (WT) or Holt-Oram mutation (HOS) TBX5 (right).
F. Uniform manifold approximation and projection for dimension reduction (UMAP) of the left ventricle (LV) snRNA-seq showing broad cluster cell types (left), and feature plots for the three genes identified by genetics as potential concern: *TBX5*, *ZNF469*, and *DNDH1*. TBX5 and DNHD1 are both predominantly expressed in the cardiomyocytes, while to a lesser degree expression is found in fibroblasts and other cell types. *ZNF469* was rarely detected.
G. Gene ontology (GO) terms representing all differentially expressed genes found comparing the HOS and control cardiomyocytes. Genes are split into those up- and down-regulated with respect to the HOS patient.
H. Odd's ratio by Fisher's exact test comparing the overlap of down- and up-regulated genes identified in **Figure 1C** and published *Tbx5* knockout (KO) mouse experiments (15-17). False discovery rate (FDR) includes comparisons made with human iPSC (**Figure 1D**).

I. Odd’s ratio by Fisher’s exact test comparing the overlap of down- and up-regulated genes identified in **Figure 1C** and published adult dilated cardiomyopathy (aDCM), pediatric dilated cardiomyopathy (pDCM), and pediatric hypertrophic cardiomyopathy (pHCM) datasets (11, 13).

Supplemental Table 1.

Critical Trio-Whole Exome Sequencing Results: Table of pathogenic/likely pathogenic variants and variants of unknown significance reported on the genetic testing.

Supplemental Table 2.

All differentially expressed genes identified by comparing the left ventricular cardiomyocytes of the HOS and control samples. The column TBX5ChIP_Distance refers to whether there was an association based on distance to nearest promoter of published TBX5 ChIP-seq from iPSC-derived cardiomyocytes (18). The column TBX5ChIP_pcHiC refers to whether there was an association based on promoter-capture Hi-C (19) and the published TBX5 ChIP-seq. The column TBX5_BindingUnion refers to whether it was identified in either the distance-based approach or the promoter-capture Hi-C. Lastly, the columns entitled MouseAtria (16), MouseVentricle (15), HumaniPSC (14), and Tbx5G125R (17) refer to whether the gene was up-regulated or down-regulated in those datasets.

Supplemental References

1. Tan C, et al. Bi-allelic variants in DNHD1 cause flagellar axoneme defects and asthenoteratozoospermia in humans and mice. Am J Hum Genet. 2022;109(1):157–171.
2. Liu S, et al. LOF variants identifying candidate genes of laterality defects patients with congenital heart disease. PLoS Genet. 2022;18(12):e1010530.
3. Abu A, et al. Deleterious mutations in the Zinc-Finger 469 gene cause brittle cornea syndrome. Am J Hum Genet. 2008;82(5):1217–1222.
4. Rohrbach M, et al. ZNF469 frequently mutated in the brittle cornea syndrome (BCS) is a single exon gene possibly regulating the expression of several extracellular matrix components. Mol Genet Metab. 2013;109(3):289–295.
5. Dickinson ME, et al. High-throughput discovery of novel developmental phenotypes. Nature. 2016;537(7621):508–514.
6. Groza T, et al. The International Mouse Phenotyping Consortium: comprehensive knockout phenotyping underpinning the study of human disease. Nucleic Acids Res. 2023;51(D1):D1038–D1045.
7. Papaioannou VE. The T-box gene family: emerging roles in development, stem cells and cancer. Development. 2014;141(20):3819–3833.
8. Abramson J, et al. Accurate structure prediction of biomolecular interactions with AlphaFold 3. Nature. 2024;630(8016):493–500.
9. Luna-Zurita L, et al. Complex interdependence regulates heterotypic transcription factor distribution and coordinates cardiogenesis. Cell. 2016;164(5):999–1014.
10. Pradhan L, et al. Intermolecular interactions of cardiac transcription factors NKX2.5 and TBX5. Biochemistry. 2016;55(12):1702–1710.
11. Hill MC, et al. Integrated multi-omic characterization of congenital heart disease. Nature. 2022;608(7921):181–191.
12. Sim CB, et al. Sex-Specific Control of Human Heart Maturation by the Progesterone Receptor. Circulation. 2021;143(16):1614–1628.
13. Koenig AL, et al. Single-cell transcriptomics reveals cell-type-specific diversification in human heart failure. Nat Cardiovasc Res. 2022;1(3):263–280.
14. Kathiriya IS, et al. Modeling Human TBX5 Haploinsufficiency Predicts Regulatory Networks for Congenital Heart Disease. Dev Cell. 2021;56(3):292–309.e9.
15. Bersell KR, et al. Transcriptional Dysregulation Underlies Both Monogenic Arrhythmia Syndrome and Common Modifiers of Cardiac Repolarization. Circulation. 2023;147(10):824–840.

16. Nadadur RD, et al. Pitx2 modulates a Tbx5-dependent gene regulatory network to maintain atrial rhythm. Sci Transl Med. 2016;8(354):354ra115.
17. van Ouwerkerk AF, et al. Patient-specific TBX5-G125R variant induces profound transcriptional deregulation and atrial dysfunction. Circulation. 2022;145(8):606–619.
18. Ang Y-S, et al. Disease Model of GATA4 Mutation Reveals Transcription Factor Cooperativity in Human Cardiogenesis. Cell. 2016;167(7):1734–1749.e22.
19. Montefiori LE, et al. A promoter interaction map for cardiovascular disease genetics. Elife. 2018;7. https://doi.org/10.7554/eLife.35788.
20. Li X, et al. The macrophage landscape across the lifespan of a human cardiac allograft. Circulation. 2024;149(21):1650–1666.
21. Wolock SL, Lopez R, Klein AM. Scrublet: Computational Identification of Cell Doublets in Single-Cell Transcriptomic Data. Cell Syst. 2019;8(4):281–291.e9.
22. Xu C, et al. Probabilistic harmonization and annotation of single-cell transcriptomics data with deep generative models. Mol Syst Biol. 2021;17(1):e9620.
23. Litviňuková M, et al. Cells of the adult human heart. Nature. 2020;588(7838):466–472.
24. Hao Y, et al. Integrated analysis of multimodal single-cell data. Cell. 2021;184(13):3573–3587.e29.
25. Zhu LJ, et al. ChIPpeakAnno: a Bioconductor package to annotate ChIP-seq and ChIP-chip data. BMC Bioinformatics. 2010;11:237.
26. Zhu LJ. Integrative analysis of ChIP-chip and ChIP-seq dataset. Methods Mol Biol. 2013;1067:105–124.
27. Zhou Y, et al. Metascape provides a biologist-oriented resource for the analysis of systems-level datasets. Nat Commun. 2019;10(1):1523.
28. Uhlén M, et al. Proteomics. Tissue-based map of the human proteome. Science. 2015;347(6220):1260419.
29. Thul PJ, et al. A subcellular map of the human proteome. Science. 2017;356(6340). https://doi.org/10.1126/science.aal3321.
