# Supplementary figures and images for: Single-nuclei transcriptomics reveals TBX5-dependent targets in a patient with Holt-Oram syndrome
Source: J Clin Invest. 2024 Nov 14;135(2):e180670. doi: 10.1172/JCI180670 (PMC11735084; doi:10.1172/JCI180670)

WCL- anti-GAPDH

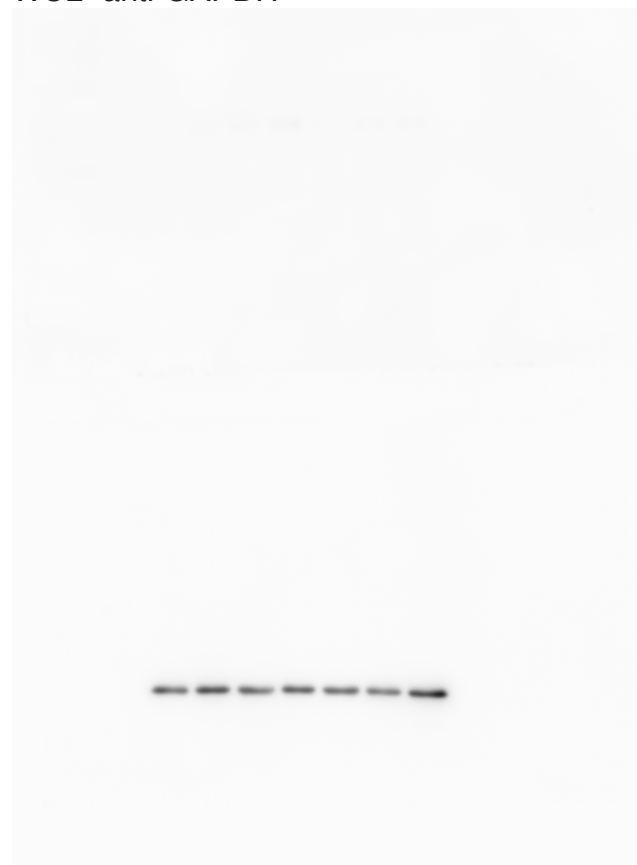

WCL- anti-HA

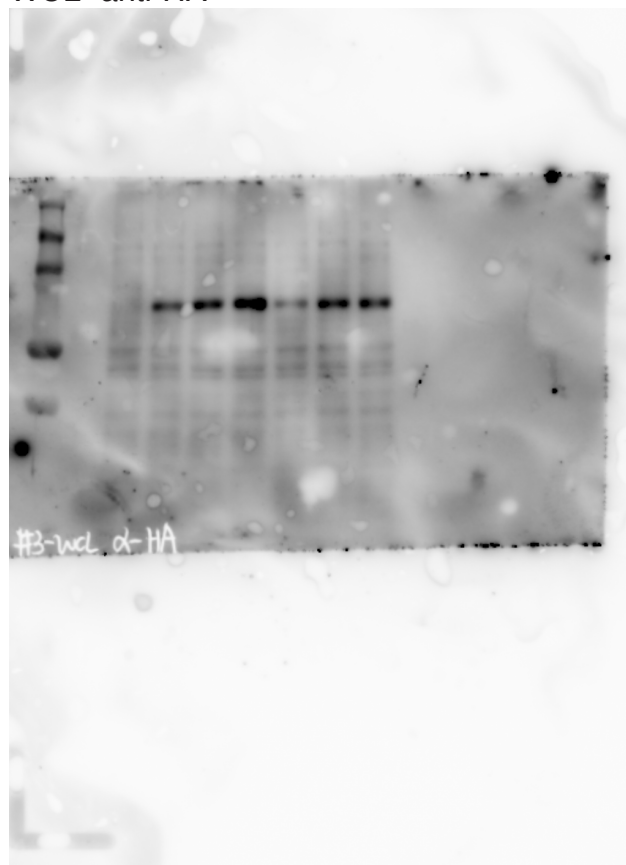

IP- anti-HA

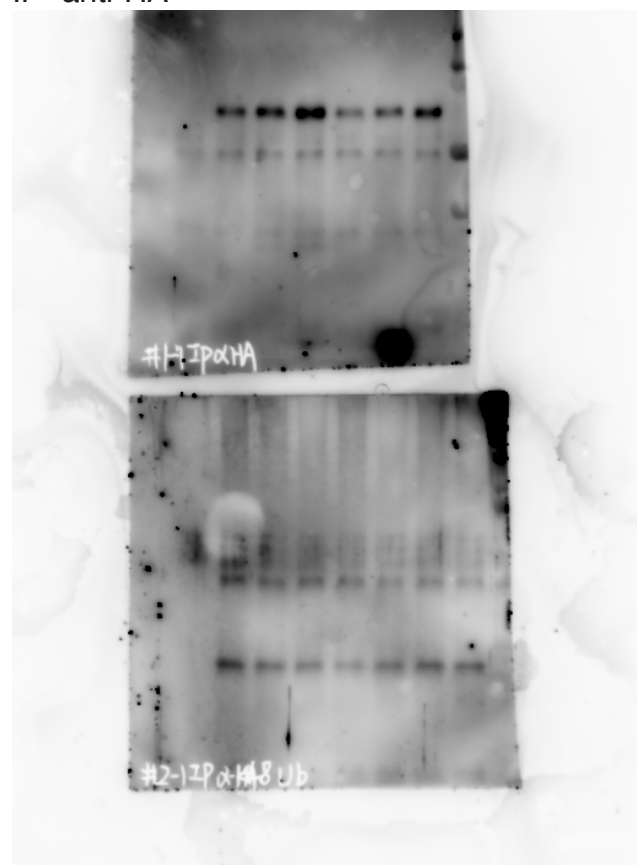

Bottom blot is not presented in this manuscript

Supplement: Unedited blot and gel images [file jci-135-180670-s123.pdf]
